# Endocardial radiofrequency ablation for the treatment of pediatric hypertrophic cardiomyopathy: A report of two cases and a brief review of the literature

**DOI:** 10.3892/mi.2026.329

**Published:** 2026-06-23

**Authors:** Fan Zhang, Bo Wang, Ye Chen, Wenjuan Shu, Jie Huang, Ling Sun

**Affiliations:** Department of Cardiology, Children's Hospital of Soochow University, Suzhou, Jiangsu 215000, P.R. China

**Keywords:** endocardial radiofrequency ablation, hypertrophic cardiomyopathy, pediatric, LZTR1 gene variant, left ventricular outflow tract obstruction

## Abstract

The present case report evaluated the application and outcomes of endocardial radiofrequency ablation (ERFA) in children with hypertrophic cardiomyopathy (HCM). The clinical data, procedural details and short-term follow-up of two pediatric patients with HCM admitted to the department of cardiology from 2023 to 2024. In addition, literature published between 2012 and 2025 was reviewed using the key words ‘endocardial radiofrequency ablation’, ‘children’ and ‘hypertrophic cardiomyopathy’ to summarize the current status and efficacy of ERFA in pediatric HCM. The first reported case was that of a 9-year-old boy who presented with reduced exercise tolerance for 6 months. An echocardiography revealed asymmetric hypertrophy of the interventricular septum and left ventricular wall, positive systolic anterior motion (SAM) sign and left ventricular outflow tract (LVOT) obstruction. Genetic testing indicated variants in *LZTR1*, suggestive of Noonan syndrome type 10. Despite medication (metoprolol and captopril), LVOT obstruction progressed (gradient >50 mmHg). ERFA was performed, and follow-up at 2 months showed reduced septal thickness and alleviated LVOT obstruction. The second case was that of an 11-year- and -11-month-old girl who presented with 3 years of dizziness and fatigue. An echocardiography confirmed asymmetric left ventricular hypertrophy, LVOT obstruction and SAM sign. Post-ERFA, LVOT obstruction significantly improved. Literature reports on ERFA for pediatric HCM are limited. Combined with adult series, ERFA appears safer than surgical myectomy or alcohol septal ablation, although long-term efficacy requires further validation. On the whole, the present case report demonstrates that ERFA is a safe and effective intervention for pediatric HCM when pharmacological therapy fails, providing a viable minimally alternative.

## Introduction

Hypertrophic cardiomyopathy (HCM) is a heterogeneous group of inherited heart diseases characterized by asymmetric myocardial hypertrophy, which compromises cardiac structure and function, and serves as a common cause of sudden death in children and adolescents (1). While the pharmacological management of pediatric HCM has been extensively discussed (2), reports on surgical interventions remain limited.

The present case report evaluated the application and outcomes of endocardial radiofrequency ablation (ERFA) in children with HCM. The clinical data, procedural details and short-term follow-up of two pediatric patients with HCM admitted to the department of cardiology from 2023 to 2024. In addition, a literature search was conducted on PubMed using the key words ‘endocardial radiofrequency ablation’, ‘children’ and ‘hypertrophic cardiomyopathy’ to summarize the current application and outcomes of endocardial radiofrequency ablation in pediatric HCM.

## Case report

The present case report evaluated the application and outcomes of ERFA in children with HCM.

The study was approved by the Medical Ethics Committee of Soochow University Children's Hospital (Approval no. 2023CS141), and informed consent was obtained from the guardians of the patients.

### Case 1

A 9-year-old boy presented to the Children's Hospital of Soochow University (Suzhou, China) with a 6-month history of decreased exercise tolerance. Initial outpatient assessment (January 4, 2023) revealed a grade II precordial systolic murmur. An echocardiography suggested HCM. Genetic variants were verified by Sanger sequencing. Target fragments were amplified by PCR and cloned into pMD19 T plasmid (Takara Bio, Inc.). Sequencing was carried out using the BigDye Terminator v3.1 kit on an ABI 3730xl DNA Analyzer according to manufacturer's protocols. Genetic testing identified two *LZTR1* variants (intron11 c.1261-2A>C, likely pathogenic; exon15 c.1648G>A, uncertain significance), indicating possible Noonan syndrome type 10. Medication with metoprolol (15 mg bid) and captopril (12.5 mg tid) was initiated.

A follow-up echocardiography in March, 2023 revealed worsening left ventricular outflow tract (LVOT) obstruction, with a pressure gradient exceeding 50 mmHg. The patient subsequently underwent ERFA under general anesthesia on March 20, 2023.

For the procedure, vascular access was obtained via the right femoral artery and left femoral vein. A significant pre-ablation LVOT gradient of 30 mmHg was recorded. Using the Carto3 system for 3D electroanatomic mapping, radiofrequency energy (40 W, 38-40˚C) was applied to the hypertrophied septal segment over approximately 20 points. Post-ablation, the LVOT gradient was significantly reduced to 5 mmHg. Immediate post-procedural echocardiography confirmed a diminished systolic anterior motion (SAM) sign, reduced septal motion, a maximal LVOT velocity of 2.0 m/sec and a gradient of 16 mmHg.

The patient remains stable at follow-up, asymptomatic and continues on medical therapy while attending school normally. The imaging data for case 1 are presented in Figs. 1, 2 and 3. The pre-operative and post-operative clinical data of the patient are summarized in Table I.

### Case 2

An 11-year-11-month-old girl presented to the Children's Hospital of Soochow University with a 3-year history of dizziness and fatigue, exacerbated by emotional triggers and occasionally accompanied by syncope. A physical examination revealed a grade III systolic murmur. Prior treatment with metoprolol and captopril was limited by poor adherence.

A pre-operative echocardiography demonstrated asymmetric septal hypertrophy with marked LVOT obstruction: A maximal flow velocity of 4.6 m/sec and a pressure gradient of 74 mmHg, along with a prominent SAM sign. In July, 2024, the patient underwent ERFA under general anesthesia.

For the procedure, vascular access was established via the right femoral artery and vein. Pre-ablation pressure measurements revealed a gradient of 76 mmHg (LV 150/20 mmHg vs. aorta 74/47 mmHg). Under CARTO3 system for 3D electroanatomic mapping (Biosense Webster, Inc.; Johnson & Johnson MedTech) guidance, ~30 ablation lesions were delivered to the hypertrophied septum at 40W, 38-40˚C. Post-procedure, the gradient decreased to 58 mmHg (LV 140/24 mmHg vs. aorta 82/56 mmHg). An echocardiography confirmed reduced SAM sign and hyperechoic changes at the ablation site.

The symptoms of the patient markedly improved during follow-up, with no further syncope or significant dizziness reported. The imaging data for case 2 are illustrated in Figs. 3 and 4. The pre-operative and post-operative clinical data of patient are summarized in Table II.

## Discussion

A literature search was conducted on PubMed using the key words ‘surgical treatment’, ‘children’ and ‘hypertrophic cardiomyopathy’. The retrieved studies, summarized in Table III, primarily focused on surgical interventions, such as the modified Morrow procedure (septal myectomy). However, there is limited literature available on endocardial radiofrequency ablation for pediatric HCM.

HCM is an autosomal dominant disorder characterized primarily by asymmetric myocardial hypertrophy and cardiac dysfunction, commonly identified in children and young adults. Clinical manifestations include dyspnea, chest pain, syncope and arrhythmias (1,3). In pediatric and adolescent populations, HCM can impair growth, lead to cardiac insufficiency and increase the risk of sudden cardiac death. The condition often demonstrates familial aggregation, with numerous mutations identified in genes encoding sarcomeric proteins (4-6). To date, >2,000 mutations in genes encoding these proteins have been associated with HCM, alongside various mitochondrial and modifier genes (3,7).

Treatment strategies for HCM include pharmacological and surgical interventions. Pharmacotherapy aims to alleviate symptoms and prevent complications. Current medications include beta-blockers, calcium channel blockers, antiarrhythmic agents, and novel drugs such as Aficamten (8,9). In the two pediatric cases discussed herein, the oral administration of metoprolol combined with captopril failed to adequately relieve outflow tract obstruction. Therefore, ERFA was performed for the treatment of HCM.

Surgical intervention for HCM aims to relieve LVOT obstruction caused by the hypertrophied myocardium, thereby improving cardiac structure and function and preventing complications (10,11). Notably, septal myectomy remains the current gold standard for the treatment of obstructive HCM.

Established surgical approaches include septal myectomy (SM), alcohol septal ablation (ASA), ERFA, heart transplantation and pacemaker implantation. As the current gold standard for obstructive HCM, septal myectomy, along with ASA, is generally safe and effective for most patients. However, potential post-operative complications include sustained ventricular tachycardia or fibrillation, and some patients may require permanent pacemaker implantation (12). Heart transplantation represents a definitive treatment for HCM. Nevertheless, its application is severely limited due to donor scarcity, significant procedural risks and the necessity for lifelong immunosuppressive medication (13). ERFA, as a minimally invasive and investigational alternative to the gold standard septal myectomy, is not a superior treatment modality compared with septal myectomy.

ERFA is a minimally invasive therapeutic approach for hypertrophic cardiomyopathy. It utilizes high-frequency electrical energy to ablate septal myocardium, thereby reducing hypertrophy and relieving left ventricular outflow tract obstruction. This technique has been widely adopted in adult populations with promising clinical outcomes. In a previous study comparing ASA and ERFA, both ASA and ERFA were shown to be viable strategies for septal reduction in HCM (14). However, ASA appeared to be more effective in achieving significant anatomic and functional improvements, whereas ERFA is excellent for avoiding bundle branch block (14). A previous meta-analysis comparing the outcomes and safety of ERFA and SM for HCM demonstrated that ERFA is a less invasive alternative to SM, but it should be characterized as an investigational alternative rather than a superior or routine replacement for the gold standard septal myectomy (15). Before obtaining more evidence from randomized controlled trials, ERFA can serve as an alternative option for pediatric patients who are at high surgical risk or unfit for septal myectomy. However, reports on the application of ERFA specifically in pediatric patients with HCM remain exceedingly limited in the literature.

ERFA effectively reduces septal and left ventricular free wall thickness, improves diastolic function and alleviates cardiac load in children with HCM, thereby mitigating symptoms and potentially reducing the risk of sudden cardiac death (16,17). At the Children's Hospital of Soochow University, the post-operative recovery period for children undergoing ERFA was notably shorter compared to conventional surgery, with minimal surgical trauma. During the 2-month follow-up after discharge, symptoms such as dyspnea, fatigue and palpitations gradually improved. Follow-up echocardiography demonstrated reduced myocardial hypertrophy and significant mitigation of LVOT obstruction. However, as an investigational alternative to the gold standard septal myectomy, ERFA carries potential risks, including cardiac perforation, atrioventricular block and arrhythmias. Given the complex etiology and heterogeneous pathophysiology of HCM, treatment outcomes can vary significantly among individuals. Therefore, further multi-center studies are essential to refine the indications for ERFA in children and to thoroughly evaluate its long-term efficacy and safety.

In conclusion, ERFA is a significant therapeutic option for pediatric HCM, which contributes to symptom relief and may reduce the risk of sudden cardiac death. When selecting this procedure, patient age and individualized procedural risks should be carefully evaluated. Optimal treatment outcomes usually require combining ERFA with other modalities, such as pharmacotherapy and cardiac rehabilitation. Notably, long-term follow-up data are necessary to assess the durability of the ablation effect, as current short-term evidence is insufficient to confirm its long-term efficacy in pediatric patients.

## Figures and Tables

**Figure 1:**
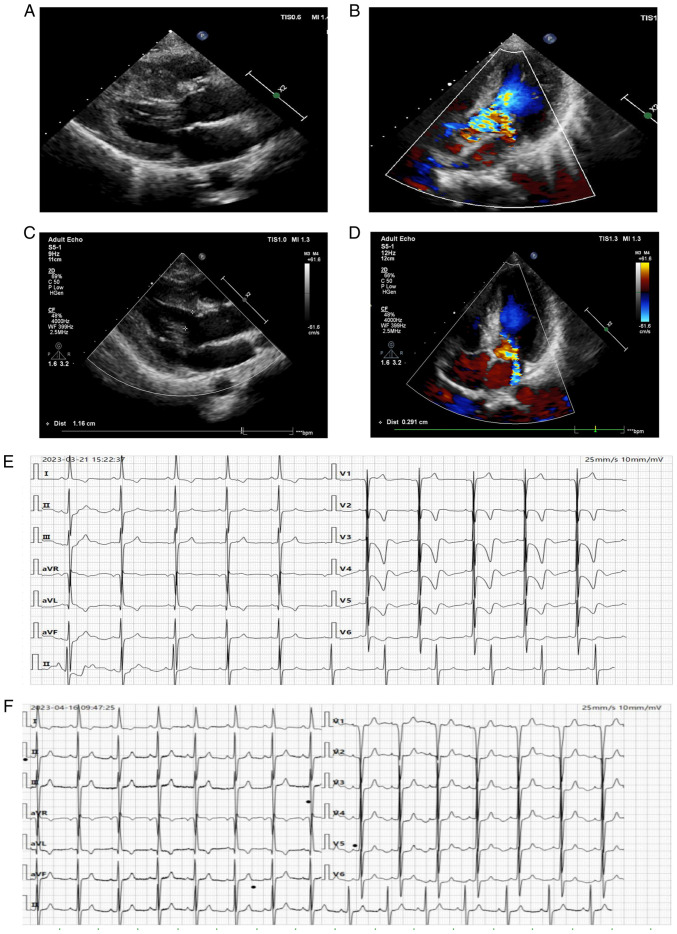
Echocardiographic and electrocardiographic findings for case 1. (A and B) Pre-operative echocardiographic images demonstrating asymmetric hypertrophy of the interventricular septum and left ventricular wall, with positive SAM of the mitral valve. The LVOT measured ~6.3 mm at its narrowest point, with a peak velocity of ~4 m/sec, corresponding to a gradient of 65 mmHg. (C and D) Post-operative (2-month follow-up) echocardiographic views demonstrating the resolution of the SAM phenomenon. The minimal LVOT diameter increased to 11.9 mm, with a reduced peak velocity of ~2.1 m/sec, corresponding to a significantly lower gradient of 18 mmHg. (E) Pre-operative and (F) 2-month post-operative electrocardiogram tracings. LVOT, left ventricular outflow tract; SAM, systolic anterior motion.

**Figure 2:**
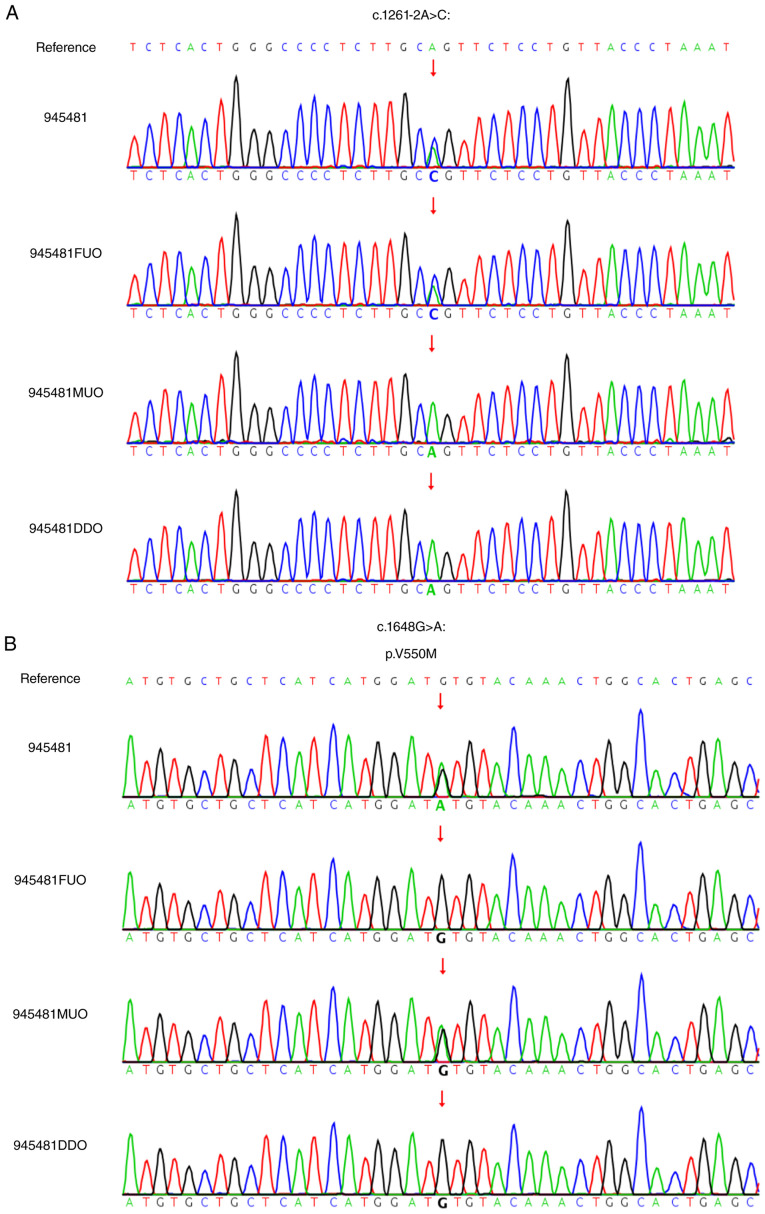
Genetic testing results for case 1. (A) An adenine was substituted by cytosine at the second base of the 3' end of position 1261 in intron 11 of the *LZTR1* gene. This variant was classified as ‘likely pathogenic’ according to the ACMG guidelines (29). (B) A guanine-to-adenine substitution at position 1648 in exon 15 of the *LZTR1* gene resulted in a missense mutation. It was rated as a ‘variant of uncertain significance’ based on the ACMG guidelines. Genetic variants were verified by Sanger sequencing. Target fragments were amplified by PCR and cloned into pMD19-T plasmid. Sequencing was carried out with BigDye Terminator v3.1 kit on an ABI 3730xl DNA Analyzer according to the manufacturer's protocols.

**Figure 3:**
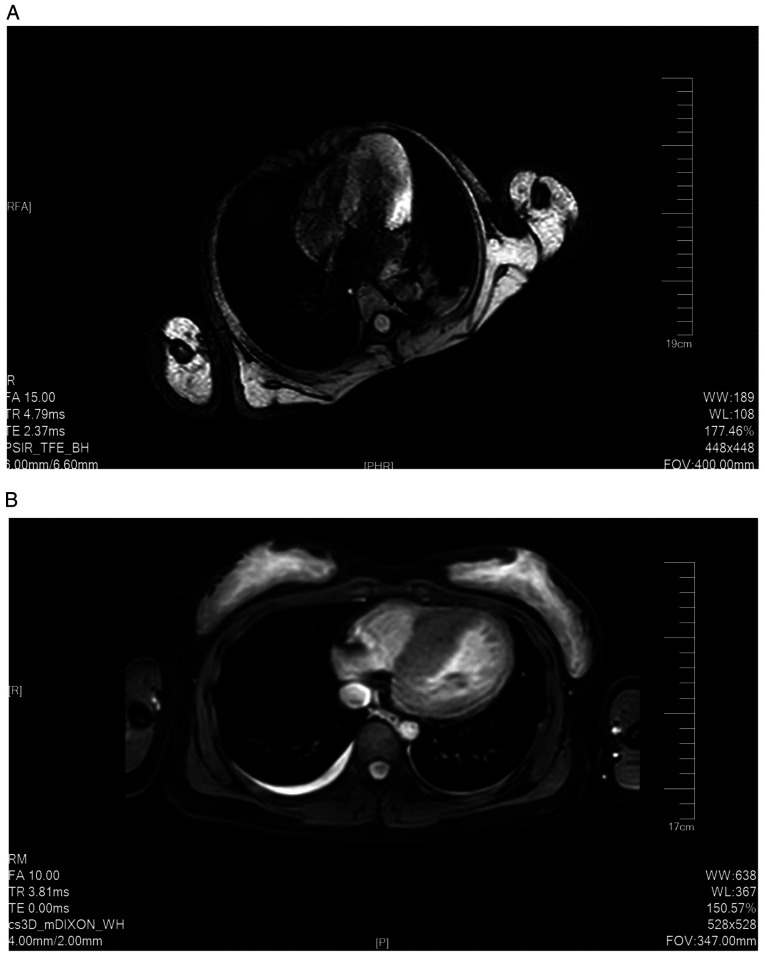
Cardiac MRI reports of the two pediatric patients. (A and B) MRI images of case 1 and case 2, respectively. The images reveal significant myocardial hypertrophy in both pediatric cases.

**Figure 4:**
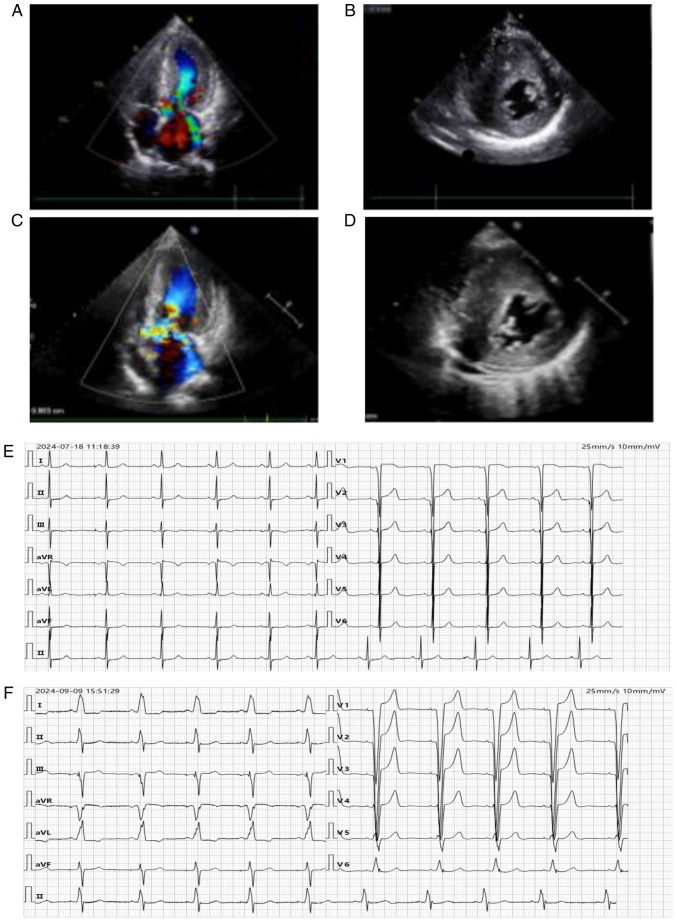
Echocardiographic and electrocardiographic findings for case 2. (A and B) Pre-operative echocardiograms demonstrate asymmetric left ventricular hypertrophy and systolic anterior motion of the mitral valve. The LVOT measured ~6.8 mm at its narrowest point, with a peak velocity of 5.7 m/sec, corresponding to a gradient of 131 mmHg. (C and D) Post-operative (2-month follow-up) echocardiograms demonstrating that the minimal LVOT diameter increased to 12.5 mm. The peak velocity was reduced to 4.2 m/s, corresponding to a gradient of 72 mmHg. (E) Pre-operative and (F) 2-month post-operative electrocardiogram tracings. LVOT, left ventricular outflow tract.

**Table I:** The pre-operative and post-operative clinical data for case 1.

Parameter	Pre-operative	Post-operative, 2 months	1-year follow-up
Interventricular septal thickness (mm)	13.4	11	10.6
Left ventricular posterior wall thickness (mm)	6.3	12	15.5
Minimum LVOT diameter (mm)	6.3	11.9	11.5
LVOT peak velocity (m/sec)	4.0	2.1	2.5
LVOT pressure gradient (mmHg)	65	18	20
Mitral regurgitation severity	Mild to moderate	Not reported	Mild to moderate
Systolic anterior motion (SAM) sign	Present	Not reported	Not reported
Abnormal electrocardiogram findings	Biventricular enlargement, QT prolongation	Sinus rhythm	Sinus rhythm

LVOT, left ventricular outflow tract.

**Table II:** The pre-operative and post-operative clinical data for case 2.

Parameter	Pre-operative	Post-operative, 2 months	1-year follow-up
Interventricular septal thickness (mm)	13.4	12	10.6
Left ventricular posterior wall thickness (mm)	16	15.8	15.5
Minimum LVOT diameter (mm)	6.8	10.9	11.5
LVOT peak velocity (m/sec)	5.7	4.1	4.1
LVOT pressure gradient (mmHg)	131	68	68
Mitral regurgitation severity	Mild to moderate	Not reported	Mild to moderate
Systolic anterior motion (SAM) sign	Present	Still visible	Not reported
ECG diagnosis	Complete left bundle branch block, poor R-wave progression	Complete left bundle branch block	Complete left bundle branch block

LVOT, left ventricular outflow tract; ECG, electrocardiogram.

**Table III:** Patients with HCM treatment modalities identified in the literature.

Authors	Year of publication	Age of patients	No. of cases	Treatment	Complication	(Refs.)
Zhu *et al*	2011	11.3±4.7 years	117	Septal myectomy	Three sudden cardiac deaths	(11)
Lawrenz *et al*	2021	58.2±13 years	41	ERASH	Pericardial tamponade	(17)
Lu *et al*	2025	49 (39-58) years	1,613	Septal myectomy	-	(18)
Zhou *et al*	2022	46.9 (14.0) years	1,314	PIMSRA	Permanent right bundle branch block in 5 patients (2.5%), resuscitated ventricular fibrillation in 2 (1.0%), and septal branch aneurysm in 2(1.0%)	(19)
Liu *et al*	2018	40.73±16.66 years	15	PIMSRA	-	(20)
Xie *et al*	2024	55.7±12.5 years	76	PIMSRA	Pericardial effusion (6/76), Cardiac tamponade and ventricular fibrillation (1/76), septal branch aneurysm (1/76)	(21)
Altarabsheh *et al*	2013	12.9±5.5 years	127	Transaortic septal myectomy	New aortic valve regurgitation requiring repair (5.5%)	(22)
Sreeram *et al*	2011	10.3±4.3 years	32	ERASH	-	(23)
Xu *et al*	2016	11.3±4.3 years	40	Septal myectomy	-	(24)
Laredo *et al*	2018	8.3±4.1 years	79	Konno procedure	-	(25)
Fang *et al*	-2023	12-18 years	2/47	Transapical beating heart septal myectomy (TA-BSM) procedure.	Iatrogenic ventricular septal perforation	(26)
Chen *et al*	2022	5.00±7.48 months	10 (Noonan syndrome)	Septal myectomy	Complete heart block	(27)
Chen *et al*	2022	18.73±26.96 months	4 (non-Noonan syndrome patients)	Septal myectomy	-	(27)
Quintana *et al*	2015	13 years (2 months to 28 years)	11	Septal myectomy	-	(28)

ERASH, endocardial radiofrequency ablation of septal hypertrophy; PIMSRA, percutaneous intramyocardial septal radiofrequency ablation.

## Data Availability

Due to ethical restrictions, the raw data cannot be made publicly available. However, de-identified data may be obtained from the corresponding author upon reasonable request.
